# fMRI-Based Alzheimer’s Disease Detection Using the SAS Method with Multi-Layer Perceptron Network

**DOI:** 10.3390/brainsci13060893

**Published:** 2023-05-31

**Authors:** Aarthi Chelladurai, Dayanand Lal Narayan, Parameshachari Bidare Divakarachari, Umasankar Loganathan

**Affiliations:** 1 Department of Electronics and Communication Engineering, Sengunthar Engineering College, Tiruchengode 637205, Tamil Nadu, India; caarthi.ece@scteng.co.in; 2 Department of Computer Science Engineering, GITAM School of Technology, GITAM University, Bengaluru 561203, Karnataka, India; dnarayan@gitam.edu; 3 Department of Electronics and Communication Engineering, Nitte Meenakshi Institute of Technology, Bengaluru 560064, Karnataka, India; 4 Department of Electrical and Electronics Engineering, S.A. Engineering College, Chennai 600077, Tamilnadu, India; drumasankar@saec.ac.in

**Keywords:** Alzheimer’s disease, functional magnetic resonance imaging, Honey Badger Optimization Algorithm, Multi-Layer Perceptron, normalization technique, Superpixels

## Abstract

In the present scenario, Alzheimer’s Disease (AD) is one of the incurable neuro-degenerative disorders, which accounts for nearly 60% to 70% of dementia cases. Currently, several machine-learning approaches and neuroimaging modalities are utilized for diagnosing AD. Among the available neuroimaging modalities, functional Magnetic Resonance Imaging (fMRI) is extensively utilized for studying brain activities related to AD. However, analyzing complex brain structures in fMRI is a time-consuming and complex task; so, a novel automated model was proposed in this manuscript for early diagnosis of AD using fMRI images. Initially, the fMRI images are acquired from an online dataset: Alzheimer’s Disease Neuroimaging Initiative (ADNI). Further, the quality of the acquired fMRI images was improved by implementing a normalization technique. Then, the Segmentation by Aggregating Superpixels (SAS) method was implemented for segmenting the brain regions (AD, Normal Controls (NC), Mild Cognitive Impairment (MCI), Early Mild Cognitive Impairment (EMCI), Late Mild Cognitive Impairment (LMCI), and Significant Memory Concern (SMC)) from the denoised fMRI images. From the segmented brain regions, feature vectors were extracted by employing Gabor and Gray Level Co-Occurrence Matrix (GLCM) techniques. The obtained feature vectors were dimensionally reduced by implementing Honey Badger Optimization Algorithm (HBOA) and fed to the Multi-Layer Perceptron (MLP) model for classifying the fMRI images as AD, NC, MCI, EMCI, LMCI, and SMC. The extensive investigation indicated that the presented model attained 99.44% of classification accuracy, 88.90% of Dice Similarity Coefficient (DSC), 90.82% of Jaccard Coefficient (JC), and 88.43% of Hausdorff Distance (HD). The attained results are better compared with the conventional segmentation and classification models.

## 1. Introduction

AD is one of the leading chronic diseases, which generally affects people over 65 years [1]. AD is a cause of dementia, which leads to behavioral issues at the acute stage, memory loss, lack of time sense, loss of spatial orientation, MCI, and retrograde amnesia [2]. The common symptoms of AD are excessive fatigue, lack of mental clarity, and mental fogginess. Generally, medical images are effective in predicting AD, where the medical images are generated from different modalities [3]. The medical imaging modality is a set of methods, which are utilized for creating visual representation of interior parts of the human body [4,5]. Medical images play a crucial role in the early prediction of abnormalities and are used for both therapeutic and diagnostic purposes [6]. Currently, several medical image acquisition modalities are invented on the basis of physical principles. Specifically, several non-invasive and invasive medical imaging methods are utilized for diagnosing AD-like fMRI, Magnetic Resonance Imaging (MRI), structural Magnetic Resonance Imaging (sMRI), cerebrospinal fluids, computerized tomography, and Positron Emission Tomography (PET) [7,8,9]. The medical imaging methods assist clinicians and physicians in improving the healthcare systems for AD.

Among the available medical imaging methods, resting state fMRI is increasingly used for AD detection, because it is a useful tool for studying the brain, in terms of low-frequency fluctuation of Blood-Oxygen-Level-Dependent (BOLD) signal and alteration related to AD [10]. The resting state fMRI image is non-invasive, where it is effective in mapping AD spreads and captures the variations of blood oxygenation levels of individuals [11]. Additionally, the fMRI images are effective in measuring brain activity by detecting the changes associated with blood flow [12]. In recent decades, numerous machine-learning approaches have been implemented for diagnosing AD. The machine-learning approaches are efficient in identifying and classifying the resting state fMRI images utilizing predetermined and labeled categories. The automatic diagnosis of AD plays a crucial role in healthcare systems because timely treatment significantly decreases the mortality rate [13]. Whereas, investigating complex brain structures in fMRI images is a time-consuming and complex task [14,15]. So, a novel automated system is implemented in this manuscript for effective segmentation and classification of AD and its types in resting state fMRI images. The primary contributions are depicted below:

- Implemented a normalization technique for improving the quality of raw resting state fMRI image by adjusting its contrast. The resultant image superiorly differentiates both bright and dark regions;
- Developed SAS methods for tissue segmentation such as AD, NC, MCI, EMCI, LMCI, and SMC. The SAS method partitions the denoised resting state fMRI image into multiple segments (a set of image pixels called super-pixels). The primary objective of the SAS method is to alter the image representation into perceptual meaning;
- Performed hybrid feature extraction (combination of Gabor and GLCM features) in order to extract vectors. Introduced HBOA for optimizing the dimensions of the extracted vectors. The feature extraction and optimization significantly reduce the number of redundant vectors that decreases the model’s effort and increases the generalization steps and learning speed;
- Used MLP classifier to classify the tissues like AD, NC, MCI, EMCI, LMCI, and SMC. The MLP classifier has two main benefits in medical image classification; (i) effectively manage enormous amounts of data and (ii) resolves complex non-linear concerns. As depicted in the resulting segment, the efficacy of the presented model is zanalyzed in light of precision, HD, f1-measure, JC, accuracy, DSC, and recall.

In this manuscript, the articles on the topic “AD detection using raw resting state fMRI image” are surveyed in Section 2. The methods, results, discussion, and conclusion of the presented model are depicted in Section 3, Section 4, Section 5 and Section 6, respectively.

## 2. Literature Review

Guo and Zhang [16] presented a new framework based on deep neural networks for early detection of AD by utilizing different medical data: fMRI images and texts that include genetics, sex, and age. In this literature, the intellectual functional networks were developed based on the signal correlation of fMRI. The presented framework almost increased 25% of diagnostic accuracy related to the conventional models. In this application, the presented improved deep learning model has two main concerns such as vanishing gradient and overfitting.

Li et al. [17] have integrated a three-dimensional Convolutional Neural Network (CNN) with Long Short-Term Memory (LSTM) network for the early detection of AD. The experiments performed on the benchmark datasets confirmed the efficacy of the hybrid model by means of accuracy. On the other hand, Alorf and Khan [18] combined brain connectivity-based Graph-Convolutional Neural Network (G-CNN) with a stacked sparse autoencoder for classifying the stages of AD from resting state fMRI images. Generally, the hybridization of two deep learning models increases the time complexity of the system.

Ramzan et al. [19] integrated a transfer-learning model with a Deep Residual Neural Network (DRNN) for automated diagnosis and multi-class classification of AD stages utilizing resting-state fMRI images. In this study, different network architectures and weight initialization methods were developed for analyzing the efficacy of the DRNN model on a benchmark dataset by means of Area under Curve (AUC), f1-measure, recall, precision, and Receiver Operating Characteristic (ROC) curve. The simulation analysis indicated that the DRNN model was effective in clinical decision-making related to the traditional models. The DRNN model has a deeper network, so it consumes more time for model training and testing.

Duc et al. [20] implemented a three-dimensional CNN model for the automatic diagnosis of AD. Here, tree regression, support vector regression, linear least square regression, group-independent component analysis, and ensemble regression based on the bagging technique were implemented to predict Mini-Mental State Examination (MMSE) score for the patients with AD. In addition to this, recursive feature elimination with Support Vector Machine (SVM) was implemented for enhancing the performance of MMSE regression. The presented model faces three main problems in AD diagnosis such as class imbalance, vanishing gradient, and overfitting.

Sethuraman et al. [21] implemented a new Deep Neuro-Functional Network (DNFN) for predicting AD using fMRI images. The presented DNFN needs limited hardware resources and was computationally more effective than the existing models. Amini et al. [22] presented a new CNN architecture for diagnosing the severity of AD. The primary aim of this study was to analyze the relationship between MMSE score and fMRI images. In this study, a multi-task feature learning approach was utilized for extracting vectors. The MMSE score was computed to analyze the severity of AD categories such as severe, moderate, mild, and low. The obtained experimental results demonstrated the efficacy of the CNN architecture over the existing machine learning models, and the CNN architecture superiorly diagnoses the stages and severity of AD with maximal accuracy. The combination of CNN with clinical imaging assists clinicians in finding prognostic markers and risk factors.

Hojjati et al. [23] initially used a graph theory algorithm for characterizing the aspects of fMRI images by computing segregation and integration measures. Secondly, the subcortical and cortical measurements were computed from the fMRI images. Then, sequential feature collection and discriminative correlation analysis were employed for feature selection and reduction. The selected features were given to the SVM classifier for classifying dissimilar groups of AD patients. The numerical analysis states the potential of the presented model based on the combination of structural and functional MRI in AD prediction, but the presented model was computationally costly.

Sun et al. [24] developed a hybrid model based on the LSTM network and CNN for predicting MCI and diagnosing AD. After collecting the fMRI images, the training samples were increased by implementing an adversarial network. The collected images were fed to the hybrid model (combination of LSTM network and CNN) for automatic prediction and classification of AD. The presented hybrid model not only predicts stable and progressive MCI but also distinguishes AD from normal health conditions. The presented hybrid model was superior to the existing machine learning models but has the problems of overfitting and high time complexity. Sarraf et al. [25] developed an optimized CNN model for recognizing the stages of cognitive impairments; however, the developed optimized CNN model was computationally expensive.

Janghel and Rathore [26] presented a novel model for the early diagnosis of AD from fMRI images. Initially, image resizing and Visual Geometry Group (VGG)-16 were employed for image pre-processing and feature extraction. Then, the AD classification was performed using different machine learning models such as decision trees, k-means clustering, linear discriminant analysis, and SVM. The evaluation measures such as sensitivity, specificity, and accuracy demonstrated the effectiveness of the presented model over the existing models. The use of traditional machine learning classifiers has two major concerns in image classification, including outliers and overfitting.

Zhang et al. [27] developed a new machine-learning framework for the automatic prediction of MCI to AD. In this study, three algorithms (sparse linear regression, minimal redundancy-maximal relevance, and random subset) were utilized for feature selection. The obtained features were given to the SVM classifier for AD and MCI prediction. The integration of three feature-selection algorithms increases the complexity of the developed framework.

Odusami et al. [28] have developed a fine-tuned ResNet-18 network for the early detection of AD using MRI images. In this study, the effectiveness of the developed ResNet-18 network was validated by means of specificity, accuracy, and recall. Shi and Liu [29] initially used the Hilbert Huang transformation technique for decomposing the collected fMRI signals into several Intrinsic Mode Functions (IMFs). Secondly, Hilbert weighted frequency was employed to extract vectors from the IMFs. Finally, the obtained vectors were given to the SVM for classifying the stages of MCI. However, the SVM classifier supports only binary class classification, which was inefficient in multi-class classification.

Anter et al. [30] have integrated a neuro-fuzzy scheme with an effective swarm intelligence optimization algorithm for the recognition of MCI from resting state fMRI images. The developed optimization algorithm has a main issue of poor convergence rate. For addressing the above-mentioned concerns, a novel model is proposed for the effective detection of AD utilizing resting-state fMRI images.

## 3. Methods

**Figure 1:**
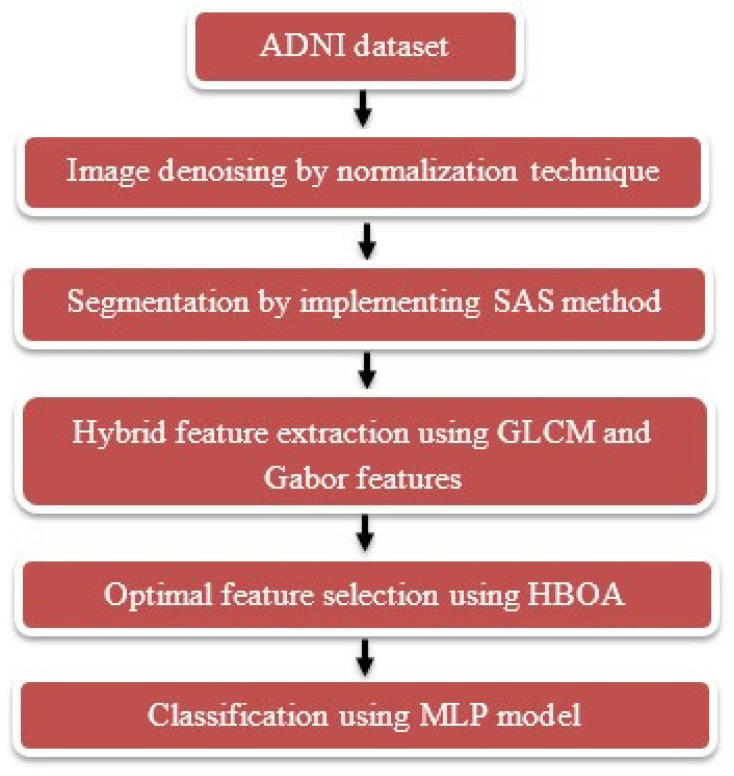
Flow diagram of the proposed system.

The proposed system includes six phases for predicting AD in resting state fMRI images such as dataset description: ADNI, denoising: normalization technique, segmentation: SAS method, feature extraction: GLCM and Gabor features, feature optimization: HBOA, and classification: MLP model. The flow diagram of the proposed system is shown in Figure 1.

### 3.1. Database Description and Denoising

In this manuscript, the proposed HBOA-MLP system’s performance is tested on a benchmark dataset named ADNI [31]. The ADNI is a multi-site study, which aims in generating clinical, neuroimaging, biochemical, and genetic biomarkers for AD tracking, prognosis, and diagnosis. The ADNI comprises neuro-images in different modalities such as Diffusion Tensor Imaging (DTI), fMRI, MRI, and PET. Here, the resting state fMRI images are utilized for experimental investigation. The collected datasets comprise 138 individuals (25 AD, 25 NC, 13 MCI, 25 EMCI, 25 LMCI, and 25 SMC) [19]. The respective individuals are labeled and diagnosed based on clinical dementia rating and MMSE score. The statistics of the ADNI dataset are mentioned in Table 1 and Table 2.

**Figure 2:**
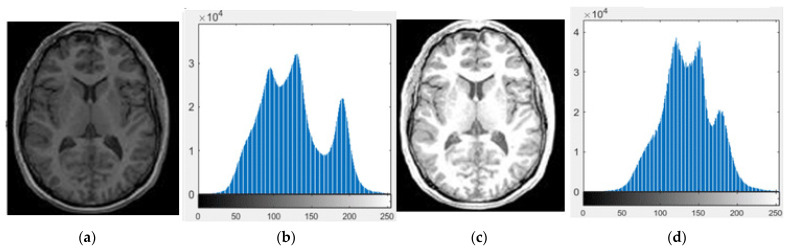
Sample resting-state fMRI images, (**a**) original image, (**b**) histogram of (**a**), (**c**) normalized image, and (**d**) histogram of (**c**).

After acquiring the resting state fMRI images, the image denoising is accomplished by using the normalization technique. In this scenario, the normalization technique improves the quality of resting-state fMRI images by altering the contrast of the images [32,33]. The sample acquired and normalized resting-state fMRI images are graphically specified in Figure 2. The formula of the normalization technique is mentioned in Equation (1). Where, the normalized fMRI image is specified as IN, the original resting state fMRI image is stated as I, minimum and maximum pixel intensity value are represented as Min and Max, which generally range between 0 and 255, and the pixel intensity value of the normalized image is mentioned as newMax and newMin.

(1) $$IN=\left(I-Min\right)+\frac{newMax-newMin}{Max-Min}+newMin$$

### 3.2. Segmentation

The segmentation process is performed once the denoised resting state fMRI image is obtained. In this scenario, the aligned super-pixels are considered for image segmentation. Diverse patterns and multi-scale visual patterns are generally used for natural images. The super-pixels have a dissimilar combination of the cues to show promising results, but not fully explored. The super-pixels are collected from the SAS for partitioning, here; bipartite graph partitioning is linear to the pixels. The resultant images are negligible and constant compared to the super-pixels [34,35]. The strong connections are relatively provided among the super-pixels and pixels of resting-state fMRI image. Steps involved in the SAS method are given below;

Input: IN is the normalized image and k is the number of segments.
Output: IS is the segmented image using k-way segmentation.

- From the image  IN, super-pixels S are collected in the bag;
- The bipartite graph is constructed;
- k Groups are derived from the bipartite graph to apply the T-cut methodology;
- The pixels are treated as the segment taken from the same group.

After the calculation of SAS, the mean orientation is determined for the extracted resting state fMRI image. The information of the objects and the regions are merged, which provides detailed information for the mean shift and resultant segmentation. The process of mean shift performs clustering, and then the data point is calculated for every data that describes the mean shift. After describing the mean shift, image segmentation, tracking, mode seeking, visual tracking, etc. are performed. The obtained data points are fed to the most important and popular estimation methodology called kernel density, where the data points are represented as i=1,…n in $R^{d}$ dimensional space, K(x) is specified as a multi-variate estimator, which is defined along with a matrix H for bandwidth, and x is indicated as computed data points, which are estimated using Equations (2) and (3).

(2) $$\hat{f}\left(x\right)=\frac{1}{n}\overset{n}{\underset{i-1}{\sum }}K_{H}\left(x-x_{i}\right)$$

where,

(3) $$K_{H}\left(x\right)={\left|H\right|}^{-\frac{1}{2}}K\left(H^{-\frac{1}{2}}x\right)$$

Hence, $K_{1}\left(x\right)$ is a symmetric univariate kernel that generates multivariate kernel functions in different ways, as shown in Equation (4).

(4) $$K^{p}\left(x\right)=\overset{d}{\underset{i=1}{\prod }}K_{1}\left(x\right)K^{S}\left(x\right)=a_{k,d}K_{1}\left(\|x\|\right)$$

where, $K^{p}\left(x\right)$ represents kernel product, which is univariate. The $K^{S}\left(x\right)$ form $K_{1}\left(x\right)$ in $\;R^{d}$, i.e., $K^{S}\left(x\right)$ is radially symmetric. The constant $a_{k,d}^{-1}=\int_{R^{d}}K_{1}\left(\|x\|\right)dx$, which assures that $K^{S}\left(x\right)$ is summed into one. The kernels are symmetric, and it is mathematically specified in Equation (5).

(5) $$K\left(x\right)=c_{k,d}k\left({\left\|x\right\|}^{2}\right)$$

where, K(x) represents kernel profile, which satisfies  x≥0, $c_{k,d}$ states the normalization constant; here $c_{k,d}\;$ makes K(x) into one by integrating and the results obtained are assumed to be positive. The Euclidean measure h for the feature space needs to be confirmed initially, and then the parameter for the bandwidth is utilized, as expressed in Equation (6).

(6) $$\hat{f}\left(x\right)=\frac{1}{nh^{d}}\overset{n}{\underset{i-1}{\sum }}K\left(\frac{x-x_{i}}{h}\right)$$

The kernel density is estimated to be superior using the mean square error that determines the optimal density. The density estimated in Equation (6) is rewritten and represented in Equation (7).

(7) $${\hat{f}}_{h,k}\left(x\right)=\frac{c_{k,d}}{nh^{d}}\overset{n}{\underset{i=1}{\sum }}K\left(\|{\frac{x-x_{i}}{h}}^{2}\|\right)$$

**Figure 3:**
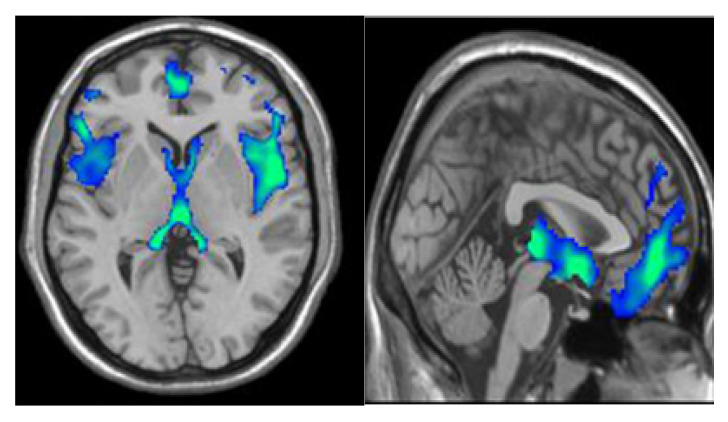
Segmentation output of the SAS method.

The original density f(x) with the feature space is initially utilized for identifying the modes of density. The gradient ∇f(x)=0 is used for locating the modes that establish zeros for the mean shift process. The gradient estimation of the density attained the estimator of gradient that employed the function linearly. In the next phase, feature extraction is carried out from the segmented tissues (AD, NC, MCI, EMCI, LMCI, and SMC) in the resting state fMRI image. The segmentation output of the SAS method is represented in Figure 3.

### 3.3. Feature Extraction

**Figure 4:**
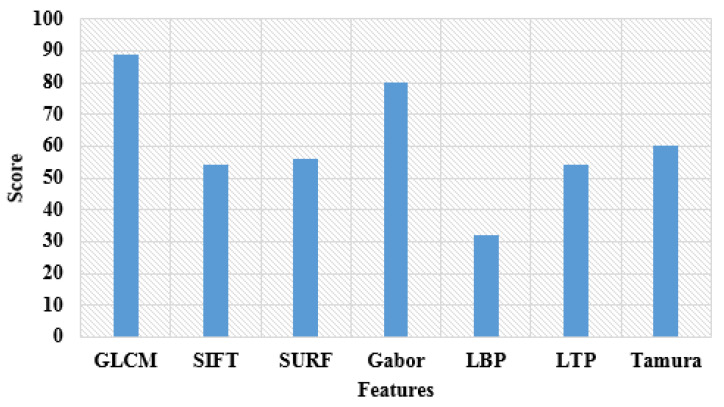
Visual analysis of feature importance score.

After segmenting the brain regions: AD, NC, MCI, EMCI, LMCI, and SMC from the denoised images, the feature extraction was performed by implementing GLCM and Gabor features. Initially, the Gabor features were calculated from the segmented fMRI images IS by utilizing the Gabor filter bank. Generally, the obtained Gabor vectors are multi-dimensional in nature and highly redundant [36]. Therefore, the Gabor filtering technique was employed for decreasing the dimensions of the extracted Gabor vectors. The extracted 2361 Gabor vectors were efficient in categorizing the brain images. The GLCM features (difference entropy, entropy, correlation, sum entropy, information measure of correlation, sum of squares, difference variance, contrast, sum average, homogeneity, sum variance, and energy) efficiently provide information about the relative neighborhood of pixel positions in the segmented resting-state fMRI images IS in order to achieve better classification results [37]. The undertaken GLCM features extract 2102 vectors from the segmented fMRI images. The extracted 4463 vectors were fed to the HBOA for dimensionality reduction, where this process decreases the processing time and complexity of the proposed system. The visual analysis of the feature importance score is stated in Figure 4.

### 3.4. Feature Optimization

The extracted 4463 vectors were fed to the HBOA for feature optimization. The HBOA follows the honey badger’s behavior to catch the prey, and this optimization algorithm has two main steps (digging and honey) for resolving the optimization problems. In the honey step, the honey badgers follow honey birds for determining the beehive. In the digging step, the prey is determined based on the smelling ability of honey badgers [38,39]. First, we initialized the agents, as shown in Equation (8).

(8) $$Z_{i}=LB_{i}+r_{1}\times \left(UB_{i}-LB_{i}\right),\;i=1,2,3,\ldots n$$

where, the lower and upper boundaries are indicated as LB and UB and the random number is represented as $\;r_{1}$, which usually ranges between zeros to one. For balancing the exploration (digging) and the exploitation (honey) searches, a density factor α is used in HBOA, and it is mathematically depicted in Equation (9).

(9) $$\alpha =l\times exp^{\left(-\frac{iter}{M_{iter}}\right)}$$

where, the maximum iteration is denoted as $\;M_{iter}$, present iteration number is specified as  iter, and the constant value is indicated as  l>1. In addition to this, a digging phase operator is employed based on the cardioid movement formula for updating the solutions, where it is mathematically expressed in Equation (10).

(10) $$Z^{new}=Z_{k}+Q\times \beta \times In\times Z_{k}+Q\times \alpha \times d^{i}\times r_{3}\times \left|cos\left(2\pi r_{4}\right)\times \left[1-cos\left(2\pi r_{5}\right)\right]\right|$$

where, β is represented as a constant value, $\;r_{3},\;r_{4},\;r_{5},\;\mathrm{and}\;r_{6}$ are denoted as a random number, $\;Z^{new}$ is indicated as the new value of $Z_{i}$ and $\;Z_{k}$, where it specifies the best solutions obtained so far. The term Q is utilized to control the search directions, as specified in Equation (11).

(11) $$Q=\{\begin{array}{c} 1,\;\;\;\;If\;\left(r_{6}\leq 0.5\right),\\ -1,\;\;\;\;\;\;\;\;\;\;\;\;\;\;\;\;Else \end{array}$$

where, the smell intensity of the prey is denoted as  In. The distance between the honey badger and the prey is computed utilizing Equations (12) and (13). As mentioned in Equation (14), the solutions are updated using the honey step operators.

(12) $$In_{i}=r_{2}\times \frac{G}{4\pi d_{i}^{2}}$$

where,

(13) $$G={\left(Z^{i}-Z^{i+1}\right)}^{2},\;d_{i}=Z_{k}-Z_{i}$$

(14) $$Z^{new}=Z_{k}+Q\times r_{7}\times \alpha \times d_{i}$$

where, the random numbers are represented as $\;r_{2}\;\mathrm{and}\;r_{7}$. As illustrated in Equation (15), the optimal vectors are selected based on $\;Fit\left(Z_{i}\right)$.

(15) $$Fit\left(Z_{i}\right)=\eta \times \frac{\left|Z_{i}\right|}{d}+ℜ\times \left(1-\gamma_{E}\left(D\right)\right),ℜ+\eta =1\;$$

where, the dependency degree is represented as $\;\gamma_{E}\left(D\right)$, which is computed utilizing Equation (16). The coefficients η and  ℜ are utilized for balancing the exploration and exploitation abilities, and d indicates optimal vectors. The term $POS_{C}\left(D\right)$ is represented as a positive region, which is mathematically denoted in Equation (17).

(16) $$\gamma_{C}\left(D\right)=\frac{\left|POS_{C}\left(D\right)\right|}{\left|U\right|}$$

(17) $$POS_{C}\left(D\right)=U\bar{K}\left(Z\right),\;Z\in \frac{U}{D}$$

where, $\bar{K}\left(Z\right)$ indicates lower approximation, and $\gamma_{E}\left(D\right)$ denotes approximating power. The parameters considered for HBOA are random numbers range is 0.6, dimension is four, β is one, maximum iteration is 100, α is one, and population size is equal to extracted vectors. From the 4463 vectors, HBOA selects 3260 vectors, which are given to the MLP model for classifying the AD and its types. The flowchart of HBOA is graphically shown in Figure 5. After feature optimization, the correlation between the selected feature vectors was analyzed by implementing Pearson’s correlation method. The correlation analysis helps in finding the association between the class features and the continuous feature vectors. Here, the probability of Pearson’s correlation value r was 0.05, and it showed that the selected feature vectors were statistically significant.

### 3.5. Classification

After the selection of 3260 vectors, MLP was employed for classifying the AD and its types such as AD, NC, MCI, EMCI, LMCI, and SMC. The MLP is one of the effective feed-forward neural networks, which includes benefits such as easy implementation and requiring only a small training set [40,41]. Generally, the MLP model has three layers such as output, hidden, and input layers. In the hidden layer, the model with an excessive or insufficient number of neurons leads to overfitting and bad generalization problems. In this research manuscript, the MLP model was implemented with three hidden layers of 10 hidden neurons. In the hidden layer, every neuron was summed with selected vectors $v_{i}$ with connection weight $\;w_{ij}$. The output of every neuron $o_{j}$ is mathematically described in Equation (18).

(18) $$o_{j}=A\left(\sum w_{ij}\times v_{i}\right)$$

where, the activation function is represented as  A, here, the hyperbolic tangent function is utilized as an activation function. The sum of square difference between the actual and the desired values of the output neurons E is mathematically expressed in Equation (19).

(19) $$E=\frac{1}{2}\underset{j}{\sum }{\left(o_{dj}-o_{j}\right)}^{2}$$

where, $\;o_{j}$ represents the actual output value of the neuron and $o_{dj}$ indicates desired output value of the neuron. Further, the weight value $w_{ij}$ is iteratively adjusted for minimizing E based on the adopted training algorithm. In this manuscript, the back-propagation of the MLP model is supported by the levenberg marquardt algorithm. The parameters assumed in the MLP model are as follows: the learning rate was 0.0001, the number of hidden layers was three, the number of hidden nodes in each layer was ten, and the activation function was a hyperbolic tangent function. The results and discussion of the segmentation method (SAS) and classification model (HBOA-MLP) are specified in Section 4.

## 4. Results

The presented segmentation method (SAS) and classification model (HBOA-MLP) were executed on a Matlab 2022a environment with a system configuration of 128 GB RAM, Windows operating system, 4 TB hard-disk, and Intel Core i9 14th-generation processor. Here, the presented SAS method and HBOA-MLP model’s performance were tested on an online dataset: ADNI by means of JC, HD, DSC, f1-measure, recall, precision, and accuracy. Particularly, the SAS method’s efficacy was analyzed in light of JC, HD, and DSC.

### 4.1. Performance Measures

The DSC was utilized for comparing the pixel-wise agreements between the ground-truth images and the segmented images. On the other hand, the JC is one of the effective functions that superiorly estimates the similarity measure between two resting-state fMRI images. In addition to this, HD is a useful and informative evaluation measure used extensively in medical image segmentation. The mathematical formulas of JC, HD, and DSC are mentioned in Equations (20)–(22).

(20) $$JC=\frac{\left|GT\cap SE\right|}{\left|GT\cup SE\right|}\times 100$$

(21) $$HD\left(GT,SE\right)=max\left\{\underset{gt\in GT}{sup}\;\underset{se\in SE}{inf}\;d\left(gt,se\right),\underset{se\in SE}{sup}\;\underset{gt\in GT}{inf}\;d\left(gt,se\right)\right\}$$

(22) $$DSC=2\frac{\left|GT\cap SE\right|}{\left|GT\right|+\left|SE\right|}\times 100$$

where,  inf is represented as infimum, GT is indicated as the ground-truth of the resting-state fMRI image,  SE is denoted as the segmented image, and sup is denoted as the supremum. The data points present in the surfaces of SE and GT are denoted as se and  gt, and the term d(gt,se) is stated as the distance between the points se and  gt.

Correspondingly, the classification performance of the HBOA-MLP model is analyzed in light of f1-measure, recall, precision, and accuracy. The f1-measure is a combined evaluation measure, which effectively captures the trade-off associated with recall and precision values. The evaluation measure: precision is defined as the proportion of classified positive cases, which are actually the real positive values.

On the other hand, recall is defined as the proportion of actual positive classes, which are precisely classified. Lastly, accuracy is defined as the ratio of the total number of predictions to the number of correct predictions. The formulas used to compute accuracy, f1-measure, recall, and precision are given in Equations (23)–(26).

(23) $$Accuracy=\frac{True\;Positive\;\left(TP\right)+True\;Negative\;\left(TN\right)}{TP+TN+False\;Positive\;\left(FP\right)+False\;Negative\;\left(FN\right)}\times 100$$

(24) $$F1-measure=\frac{2TP}{FP+2TP+FN}\times 100$$

(25) $$Recall=\frac{TP}{TP+FN}\times 100$$

(26) $$Precision=\frac{TP}{TP+FP}\times 100$$

### 4.2. Quantitative Study Related to Segmentation

**Figure 6:**
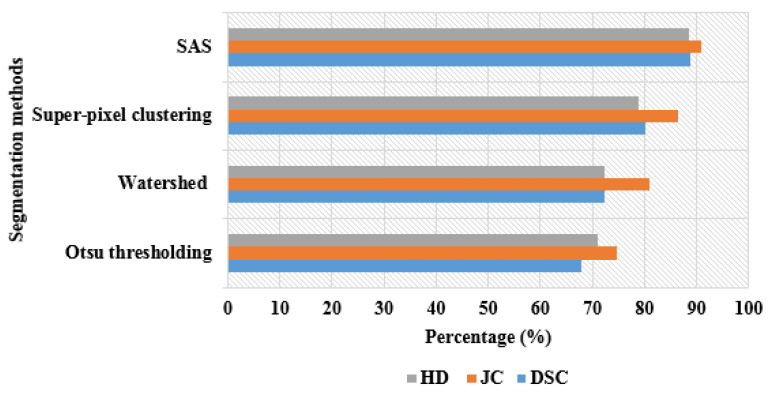
Visual presentation of the SAS method and other comparative segmentation method’s results.

In the initial phase, the quantitative results of the proposed SAS method and other comparative segmentation methods are stated in Table 3. As depicted in Table 3, the proposed SAS method has better segmentation results with DSC of 88.90%, JC of 90.82%, and HD of 88.43%. The obtained results are superiorly higher than the existing segmentation methods such as the Otsu thresholding technique, watershed algorithm, and super-pixel clustering technique. Hence, the primary objective of the SAS method is to simplify the representation of denoised resting-state fMRI images into perceptual meaning. The visual presentation of the SAS method and other comparative segmentation method results are mentioned in Figure 6.

### 4.3. Quantitative Study Related to Classification

The experimental results of the HBOA-MLP model and other comparative classification models are mentioned in Table 4. In this scenario, the classification model’s performance was analyzed without performing optimization and with HBOA. In the case without performing feature optimization, the MLP classifier obtained better classification results with an f1-measure of 90.16%, precision of 90.20%, recall of 91.90%, and accuracy of 86.50%. The results are superior to other classification models such as random forest, XGBoost, decision tree, and SVM. Compared to the existing machine learning classifiers, the MLP includes the following benefits: (i) makes faster predictions after training, (ii) efficiently manages enormous amounts of input data, (iii) resolves complex non-linear concerns, and (iv) achieves higher classification accuracy with a limited number of data samples.

**Figure 7:**
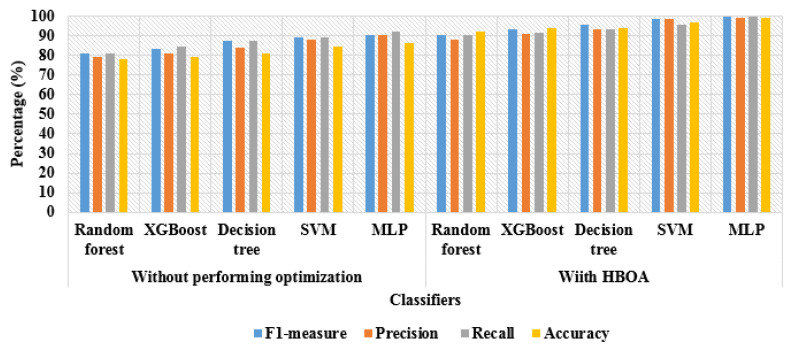
Visual presentation of HBOA-MLP model and other comparative classification model results.

On the other hand, in the case of HBOA, the MLP classifier obtained an f1-measure of 99.55%, precision of 99.28%, recall of 99.55%, and accuracy of 99.44%, which are superior to comparative classification models. Here, the experimental investigation is performed with 80:20% of training and testing of resting-state fMRI images. The visual presentation of the HBOA-MLP model and other comparative classification model results are specified in Figure 7.

**Figure 8:**
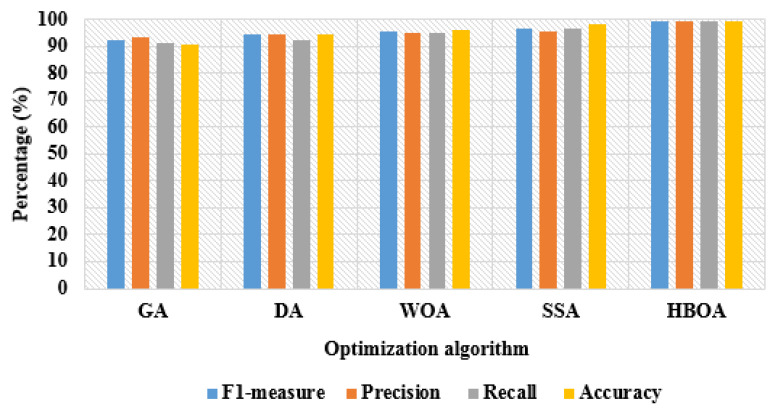
Visual presentation of HBOA and other optimization algorithm results.

The experimental results of HBOA and other optimization algorithms are stated in Table 5. Related to the comparative optimization algorithms: Genetic Algorithm (GA), Dragonfly Algorithm (DA), Whale Optimization Algorithm (WOA), and Squirrel Search Algorithm (SSA), the HBOA has higher classification results by means of f1-measure, precision, recall, and accuracy. As mentioned in the introduction section, the selection of optimal vectors significantly decreases the system complexity to linear and the computational time of image classification to 62.11 s. The proposed HBOA has a faster convergence rate than other optimization algorithms and significantly avoids local optima traps, while dealing with enormous amounts of data. The visual presentation of HBOA and other optimization algorithm results is depicted are Figure 8.

### 4.4. Comparative Study

The efficacy of the HBOA-MLP model was validated with the existing model: DRNN developed by Ramzan et al. [19], and it is specified in Table 6. In the existing literature, Ramzan et al. [19] combined transfer learning with DRNN for classifying AD stages (AD, NC, MCI, EMCI, LMCI, and SMC) using resting-state fMRI images. Here, different weight initialization methods and network architectures were used for analyzing the effectiveness of the DRNN model on a benchmark dataset: ADNI. The acquired ADNI datasets comprise 138 individuals (25 AD, 25 NC, 13 MCI, 25 EMCI, 25 LMCI, and 25 SMC). The experiments conducted on the ADNI dataset demonstrated the effectiveness of the DRNN with a mean classification accuracy of 97.84%. Compared to the existing DRNN model, the HBOA-MLP model obtained a maximum mean classification accuracy of 99.44% on the ADNI dataset using resting-state fMRI images.

## 5. Discussion

As mentioned in the introduction section, segmentation and feature optimization are integral parts of this research. The SAS significantly segments the brain regions (AD, NC, MCI, EMCI, LMCI, and SMC) from the denoised resting-state fMRI images. Further, the selection of optimal vectors decreases the system complexity to linear and the computational time of image classification to 62.11 s. However, the computational time of the HBOA-MLP model is superior to other comparative classification models and optimization algorithms. The efficacy of the presented segmentation method (SAS) and classification model (HBOA-MLP) are depicted in Table 3, Table 4, Table 5 and Table 6.

## 6. Conclusions

In this manuscript, a novel segmentation method (SAS) and classification model (HBOA-MLP) were proposed for the early diagnosis of AD using fMRI images. First, the quality of the collected fMRI images was enhanced by implementing the normalization technique, and, further, the brain regions (AD, NC, MCI, EMCI, LMCI, and SMC) were superiorly segmented by employing the SAS method. Next, the hybrid feature extraction and optimization were accomplished by utilizing Gabor features, GLCM features, and HBOA. The dimensionally reduced vectors were fed to the MLP classifier for image classification. In this scenario, the evaluation measures precision, HD, f1-measure, JC, accuracy, DSC, and recall were utilized for analyzing the efficacy of the segmentation method (SAS) and classification model (HBOA-MLP). As mentioned in the resulting segment, the HBOA-MLP model attained 99.44% of accuracy, and it is superior to the conventional comparative machine-learning models. On the other hand, the selection of optimal active vectors by HBOA diminished the proposed system complexity to linear and decreased the computational time of segmentation and classification to 42.33 s and 62.11 s. However, the MLP network includes too many parameters, because of its fully connected nature, and here, every node is connected with another node in a dense web that results in higher redundancy and inefficiency in the larger datasets. 

As a future extension, an effective transfer learning based CNN model is proposed with HBOA for precise AD prediction, because the MLP network is not ideal in processing patterns with multidimensional data. In addition, multimodal data (a combination of Electroencephalography (EEG), fMRI, and MRI) can be utilized for further enhancing the performance of AD prediction.

## Figures and Tables

**Figure 5:**
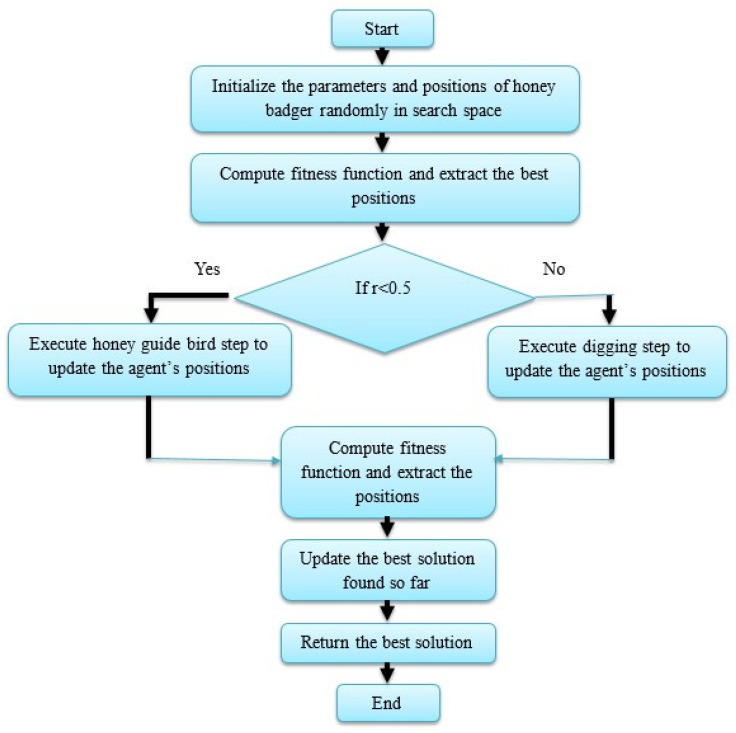
Flow chart of HBOA.

**Table 1:** Statistics of the ADNI dataset.

Properties	Description
Format	Digital imaging and communications in medicine
Slice thickness	3.31
Pixel spacing	3.31
Slices	6720
Height, width	64, 64
Flip angle	80°
Field strength	3.0
Acquisition scanner	Philips medical systems
Echo-planar imaging	140 images per volume

**Table 2:** Overview of the ADNI dataset.

Classes	Subjects	Mean Age
AD	25	74.69
NC	25	75.09
MCI	13	75
EMCI	25	71.87
LMCI	25	72.27
SMC	25	72.51
Total	138	-

**Table 3:** Results of the SAS method and other comparative segmentation methods.

Segmentation Methods	DSC (%)	JC (%)	HD (%)
Otsu thresholding	67.80	74.55	70.90
Watershed	72.33	80.90	72.39
Super-pixel clustering	80.12	86.53	78.90
SAS	88.90	90.82	88.43

**Table 4:** Results of HBOA-MLP model and other comparative classification models.

Without Performing Optimization				
Classifiers	F1-Measure (%)	Precision (%)	Recall (%)	Accuracy (%)
Random forest	81.23	79.28	81.18	77.90
XGBoost	83.39	81.26	84.30	79.45
Decision tree	87.59	83.86	87.28	81.28
SVM	88.99	88.33	89.26	84.38
MLP	90.16	90.20	91.90	86.50
**With HBOA**				
**Classifiers**	**F1-Measure (%)**	**Precision (%)**	**Recall (%)**	**Accuracy (%)**
Random forest	90.40	88.34	90.38	92.20
XGBoost	93.30	90.98	91.42	93.82
Decision tree	95.58	93.26	93.24	94.20
SVM	98.68	98.70	95.78	96.78
MLP	99.55	99.28	99.55	99.44

**Table 5:** Results of HBOA and other optimization algorithms.

Optimization Algorithm	F1-Measure (%)	Precision (%)	Recall (%)	Accuracy (%)
GA	92.38	93.36	90.93	90.59
DA	94.26	94.56	92.36	94.43
WOA	95.45	94.94	94.87	95.95
SSA	96.67	95.65	96.38	98.45
HBOA	99.55	99.28	99.55	99.44

**Table 6:** Comparative results of HBOA-MLP and DRNN.

Models	Mean Classification Accuracy (%)
HBOA-MLP	97.84
DRNN [19]	99.44

## Data Availability

The dataset used and/or analyzed during the current study are available from the corresponding author on reasonable request.
